# Emerging Epigenetic Therapeutic Targets in Acute Myeloid Leukemia

**DOI:** 10.3389/fonc.2019.00850

**Published:** 2019-09-06

**Authors:** Bettina Wingelhofer, Tim C. P. Somervaille

**Affiliations:** Leukaemia Biology Laboratory, Cancer Research UK Manchester Institute, University of Manchester, Manchester, United Kingdom

**Keywords:** AML, LSD1, EZH2, BET bromodomain, DOT1L, PRMT5, EP300, MLL

## Abstract

Acute myeloid leukemia (AML) is a genetically heterogeneous malignancy for which treatment options have been largely limited to cytotoxic chemotherapy for the past four decades. Next-generation sequencing and other approaches have identified a spectrum of genomic and epigenomic alterations that contribute to AML initiation and maintenance. The key role of epigenetic modifiers and the reversibility of epigenetic changes have paved the way for evaluation of a new set of drug targets, and facilitated the design of novel candidate treatment strategies. More recently, seven new targeted therapies have been FDA-approved demonstrating successful implementation of the past decades' research. In this review, we will summarize the most recent advances in targeted therapeutics designed for a focused group of key epigenetic regulators in AML, outline their mechanism of action and their current status in clinical development. Furthermore, we will discuss promising new approaches for epigenetic targeted treatment in AML which are currently being tested in pre-clinical trials.

## Introduction

Epigenetic regulation of the state of a cell involves various dynamic and reversible post-translational modifications of DNA and histone proteins. These modifications in their totality regulate the accessibility of DNA for the transcription machinery, thereby determining which specific genomic loci are transcriptionally active or repressed (1). The best-researched chromatin modifications include lysine acetylation, lysine mono-, di-, or tri-methylation, and arginine methylation. In addition, DNA methylation is an important regulator of gene expression and other DNA-dependent processes.

Normal hematopoiesis is regulated by the cooperative action of various transcription factors and epigenetic modulators that drive cell type-specific transcriptional programs. Recent advances in next-generation sequencing-based approaches and global projects, such as the Encyclopedia of DNA Elements (ENCODE, 2003), The Cancer Genome Atlas (TCGA, 2006), the International Cancer Genome Consortium (ICGC, 2008), and the European Community initiative BLUEPRINT (2011) have been critical in defining the regulatory networks in different normal hematopoietic cell types as well as how they are deregulated in myeloid malignancies (2–4). The key role of epigenetic modifiers in diseases, such as leukemia and the reversibility of epigenetic changes create an opportunity for development of targeted therapies with significant implications for clinical prevention and treatment. Indeed, a plethora of preclinical and clinical studies covering several hematologic malignancies show that targeting these epigenetic regulators can restore normal epigenetic and transcriptional programs (5, 6). Acute myeloid leukemia (AML) represents a group of genetically heterogeneous malignant clonal disorders which share the common feature of a block to normal myeloid differentiation. Various genetic and epigenetic mechanisms regulating the pathophysiology of AML have been identified many of which cluster in particular categories of genes including those coding for signaling molecules (such as *FLT3* and *KIT*), transcription factors (such as *CEBPA* and *RUNX1*), chromatin modifiers (such as *MLL* and *ASXL1*) or direct and indirect regulators of DNA methylation (such as *DNMT3A, IDH1, IDH2*, and *TET2*) (7, 8). Although the number of potential targets for novel therapeutics has expanded in the last decade, a major challenge in AML is the genetic heterogeneity; there remains a substantial lack of understanding as to how mutations and their associated aberrant patterns of epigenetic modification interact with one another to confer malignant transformation. Perhaps as a result, with some notable exceptions, certain clinical studies of candidate epigenetic therapies have yielded disappointing results.

Until recently, FDA-approved targeted therapies in myeloid malignancies were limited to all-trans retinoic acid (ATRA) and arsenic trioxide (ATO) for treatment of acute promyelocytic leukemia (9) and the DNA hypomethylating agents decitabine and 5-azacitidine targeting DNA methyltransferases (DNMTs) for the treatment of myelodysplasia (10, 11). However, since 2017 seven new targeted therapies have been FDA-approved in AML. These are the mutant IDH1 inhibitor ivosidenib and the mutant IDH2 inhibitor enasidenib, for patients with relapsed or refractory AML with the appropriate mutation; the BCL2 inhibitor venetoclax in combination with azacitidine or decitabine or low-dose cytarabine for newly-diagnosed AML in the elderly; the smoothened receptor inhibitor glasdegib in combination with low-dose cytarabine for newly-diagnosed AML in the elderly; gemtuzumab ozagamicin for newly-diagnosed CD33^+^ AML alone or in combination with conventional chemotherapy; the multi-kinase inhibitor midostaurin for newly diagnosed FLT3-mutated AML in combination with conventional chemotherapy; and the FLT3, AXL, and ALK inhibitor gilteritinib for relapsed or refractory FLT3-mutated AML (12–19). IDH1 and IDH2 inhibitors are excellent examples of what are presumed to be epigenetic therapies, but with an indirect mechanism of action. Through blockade of production of the putative oncometabolite D-2-hydroxyglutarate, which is a competitive inhibitor of α-ketoglutarate-dependent dioxygenases, such as the TET family of 5-methylcytosine hydroxylases, the Jumonji family of lysine demethylases and prolyl hydroxylases, they target altered transcriptional programs in AML caused by global changes in DNA methylation and histone modifications (12). As a general principle these compounds have only moderate activity as single agents (3, 20) and so research into their combinatorial use remains an intense and active area of interest. As an aside, it is worth noting that histone deacetylase (HDAC) inhibitors vorinostat and panobinostat are approved for use in cutaneous T-cell lymphoma and multiple myeloma, respectively, and that the oral HDAC inhibitor pracinostat is currently being tested in a phase 3 setting in combination with azacitidine in elderly patients with AML (NCT03151408).

While these new developments in FDA approval are welcome, there remains much to do to improve the outcome of patients with myeloid malignancies. In this perspective, we will discuss a discrete set of candidate epigenetic therapeutic targets currently under evaluation in AML: the lysine demethylase LSD1, the protein methyltransferases EZH2, DOT1L, and PRMT5, and the BET bromodomain proteins. We will describe the importance of these transcriptional activators and repressors in different AML subtypes as well as their targeting potential, possible limitations and potential toxicities. We will summarize their current status in clinical development. For detailed review of other equally important targets, such as DNMTs and HDACs the reader is referred to recent comprehensive reviews (20–22). Finally, we will discuss a number of novel epigenetic targets currently undergoing pre-clinical evaluation.

## Targeting Epigenetic Repressors in Acute Myeloid Leukemia

### LSD1

Histone methylation and demethylation are tightly regulated, dynamic processes that regulate transcriptional activation or repression depending on the location of the modification. Methylation is generated by specific histone methyltransferases (HMTs), such as MLL, DOT1L, and EZH2. As for other histone modifications, methylation can be reversed by two classes of demethylases (KDM): the larger Jumonji domain family and the smaller lysine-specific demethylase (LSD) family.

**Figure 1:**
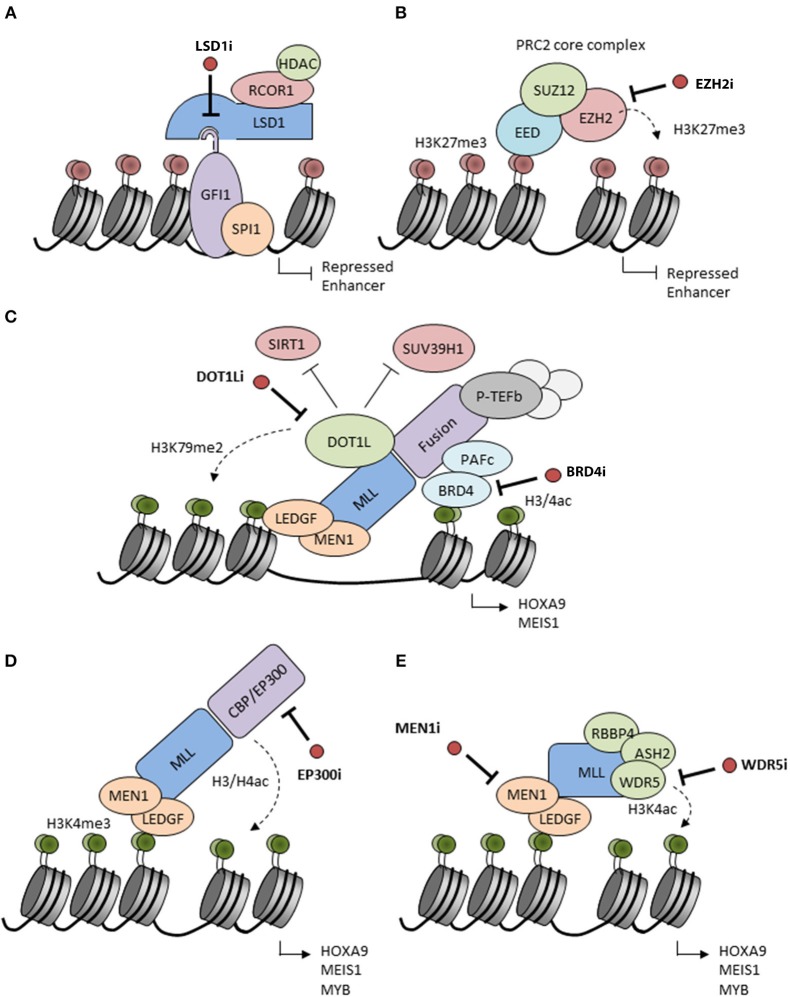
Putative mechanisms of action of candidate epigenetic inhibitors. **(A)** LSD1 interacts with the SNAG domain of GFI1 recruiting repressors to chromatin. Inhibitors of LSD1 (LSD1i) disrupt the interaction and inactivate GFI1 leading to enhancer acetylation and activation. LSD1 inhibitors also inactivate the histone demethylase activity of LSD1 (not shown). **(B)** EZH2 catalyzes H3K27 methylation inducing transcriptional repression. This activity is blocked by S-adenosyl-methionine (SAM)-competitive inhibitors of EZH2 (EZH2i). **(C)** MLL fusion proteins form complexes on chromatin with Polymerase Associated Factor complex (PAFc) (which recruits Super Elongation Complex components), Positive Transcription Elongation Factor b (pTEFb) and other factors to facilitate the expression of MLL-driven target genes, such as *HOXA9* and *MEIS1*. DOT1L is ectopically recruited by MLL fusions and adds activating H3K79me2 marks while reducing H3K9me2 repressive marks by inhibition of SUV39H1 and SIRT1. BRD4 recognizes H3K27ac marks and is essential for recruitment and stabilization of the MLL complex on chromatin. Inhibitors of the enzymatic activities of DOT1L (DOT1Li) or BRD4 (BRD4i) are considered to disrupt the MLL fusion protein complexes leading to the release of the differentiation block. **(D)** MLL may be fused to the histone acetyltransferases CBP or EP300 which are associated with H3/H4 acetylation and active gene transcription. CBP/EP300 bromodomain inhibition (EP300i) decreases H3K27 acetylation and chromatin accessibility at target promoters and enhancers. **(E)** The N-terminal part of the MLL complex associates with different proteins, such as LEDGF and Menin which stabilize the complex on chromatin. Proteins, such as RBBP5, ASH2L, and WDR5 interact with the MLL C-terminus to facilitate SET domain-mediated H3K4 methylation. Inhibition of these interactions (MEN1, WDR5i) disrupt the MLL complex and decrease expression of *HOXA9* and *MEIS1*.

LSD1/KDM1A is a flavin-adenine dinucleotide (FAD)-dependent histone demethylase (23, 24) with activity vs. mono- and dimethyl-H3K4 and H3K9 marks as well as non-histone proteins, such as DNMT1 and TP53 (25). LSD1 is typically found as a component of repressive multi-subunit complexes, such as CoREST and NuRD (26–30). More recent studies have revealed that LSD1 also binds with high affinity to N-terminal sequences of SNAG domain transcription factor family members, an interaction facilitated by molecular mimicry of the histone H3 tail by the SNAG domain (31–33). Indeed physical association of LSD1 with the SNAG domain of GFI1 is essential for the activity of GFI1 as a transcription repressor (34). In keeping with these observations, LSD1 has a critical role in normal hematopoiesis as well as in hematological malignancies (25, 35). In MLL-rearranged AMLs, LSD1 is critical for maintenance of leukemic stem cell (LSC) potential by sustaining an oncogenic transcriptional program and blocking differentiation and apoptosis (36). Inhibition of LSD1 by tranylcypromine sensitizes AML cells to differentiation induction by all-trans-retinoic acid (ATRA) (35). An essential point is that inhibitors of LSD1 both inhibit the demethylase activity of the enzyme and block the physical interaction of LSD1 with GFI1, thus impairing enzymatic and scaffolding functions of the protein, and inactivating the transcription repressor activity of SNAG domain transcription factors (Figure 1A).

In recent years, two tranylcypromine-derivative inhibitors, GSK2879552 (NCT02177812) and iadademstat (ORY-1001; EudraCT 2013-002447-29), have been evaluated in phase I trials in patients with relapsed or refractory AML (Table 1). While the former was terminated due to an unfavorable risk-benefit assessment, preliminary results are available from the latter: iadademstat was well tolerated and induced molecular and morphologic blast cell differentiation in patients harboring *MLL* gene translocations (37, 38). Preclinical data have suggested the possibility of synergistic effects of LSD1 inhibition with ATRA. As a result, tranylcypromine itself and derivatives, such as IMG-7289 are undergoing evaluation in trials in combination with ATRA (NCT02717884, NCT02273102, NCT02261779, NCT02842827, and EudraCT 2012-002154-23); results are awaited. Following on from phase 1, GSK2879552 and iadademstat are now being evaluated in combination with azacitidine in high risk myelodysplasia (NCT02929498) and AML, respectively (EudraCT 2018-000482-36).

**Table 1:** Key clinical trials of novel epigenetic therapies in AML (March 2019).

**Target**	**Drug**	**Trial number**	**Phase**	**Status**
**TRANSCRIPTIONAL REPRESSORS**				
LSD1	IMG-7289	NCT02842827	1	Completed
	GSK525762	NCT02177812	1	Terminated
	Tranylcypromine	EudraCT 2012-002154-23	1/2	Completed
		NCT02717884	1/2	Recruiting
		NCT02273102	1	Recruiting
		NCT02261779	1/2	Unknown
	ORY-1001	EudraCT 2013-002447-29	1	Completed
		EudraCT 2018-000482-36	1	Ongoing
EZH2	DS-3201b	NCT03110354	1	Recruiting
**TRANSCRIPTIONAL ACTIVATORS**				
DOT1L	EPZ-5676	NCT02141828	1	Completed
		NCT01684150	1	Completed
		NCT03724084	1/2	Recruiting
PRMT5	GSK3326595	NCT03614728	1/2	Recruiting
BET proteins	OTX015/MK-8628	NCT01713582	1	Completed
		NCT02698189	1	Active, not recruiting
	ABBV-744	NCT03360006	1	Recruiting
	RO6870810	NCT02308761	1	Completed
	PLX51107	NCT02683395	1	Terminated
	FT-1101	NCT02543879	1/1b	Recruiting
	ABBV-075	NCT02391480	1	Active, not recruiting
	CPI-0610	NCT02158858	1/2	Recruiting
	INCB054329	NCT02431260	1/2	Completed
	GSK525762	NCT01943851	1	Recruiting
		EudraCT 2013-000445-39	1/2	Ongoing
CREBB/EP300	C646103			Pre-clinical
	I-CBP112			Pre-clinical
	CCS1477			Pre-clinical
Menin	MI-463			Pre-clinical
	MI-503			Pre-clinical
WDR5	MM401			Pre-clinical
	OICR9429			Pre-clinical

### EZH2

EZH2 is the catalytic subunit of Polycomb Repressive Complex 2 (PRC2) which is responsible for maintaining transcriptional repression of its target genes through tri-methylation of H3 K27 (39, 40) (Figure 1B). This histone mark facilitates recruitment of PRC1 and ubiquitination of H2A K119 to induce a higher repressive state of chromatin (41, 42). EZH2 regulates normal hematopoiesis by maintaining multipotency and self-renewal of hematopietic stem cells (HSCs) (39, 40). However, conditional knockout studies have shown that it is dispensable for HSCs possibly because of redundancy with EZH1 (43). During the last decade, EZH2 has generated much interest as a potential anti-cancer therapeutic strategy. First, several studies have implicated PRC2 complex components including EZH2 in the pathogenesis of diverse cancers including hematopoietic malignancies (44). More recently, distinct cancer-associated mutations in EZH2 have been reported, including gain-of-function mutations in lymphoid malignancies and loss-of-function mutations in myeloid malignancies where they are also associated with a poor prognosis (42). Loss of EZH2 activity in myeloid malignancies also results from differential splicing in the presence of an SRSF2 mutation or consequent upon ASXL1 mutation (45, 46). Interestingly in certain pre-clinical studies using EZH2-deficient mouse models, EZH2 is highlighted as required for the development of myeloid malignancies including MLL-AF9 AML; mutation or deletion leads to a significant loss of LSCs and increased differentiation (47).

The greatest focus for the clinical development of EZH2 inhibitors has been in the setting of lymphoma and some solid tumors (NCT02082977, NCT01897571, NCT02395601) where S-adenosyl methionine-competitive inhibitors, such as GSK2816126, CPI1205, and the most promising, tazemetostat (EPZ-6438), have been evaluated. Another is MAK683 which is an EED-binding complex disrupter under investigation in refractory lymphoma and solid malignancies (NCT02900651). Phase I data for tazemetostat demonstrated an acceptable safety profile and some objective responses (NCT01897571) resulting in initiation of a number of follow on single agent and combination studies (e.g., NCT02875548). On the basis of pre-clinical studies demonstrating the functional importance of PRC2 in *MLL*-rearranged AML, the dual EZH1–EZH2 inhibitor DS3201b has entered phase 1 as monotherapy in patients with refractory acute leukemia (NCT03110354) (Table 1). However, given a recent report implicating loss of EZH2 and subsequent reduction of histone H3K27 trimethylation in acquired resistance to tyrosine kinase inhibitors (TKIs) and cytotoxic drugs in AML due to derepression of *HOX* genes (48), cautious selection of specific patient groups is required.

## Targeting Epigenetic Activators in Acute Myeloid Leukemia

### DOT1L

Chromosomal rearrangement of *MLL* (*KMT2A*) occurs in around 5% of AML cases, predominantly resulting in an *MLL*-*AF9* fusion gene, although other partner genes occur less frequently (49, 50). The resulting oncoprotein maintains its ability to bind to MLL target genes through N-terminal sequences but recruits additional proteins to MLL target genes through C-terminal sequences. These include members of transcriptional elongation complexes, such as the super elongation complex (SEC) and the H3K79 methyltransferase DOT1L (51).

DOT1L is the only protein methyltransferase responsible for catalyzing methylation of H3K79 (52), a modification generally associated with active transcription (53). Aberrant recruitment of DOT1L results in abnormally high levels of H3K79 methylation on promoters and gene bodies of MLL-fusion target genes, including the *HOXA* cluster and the homeobox gene *MEIS1* (54, 55), which are associated with hematopoietic transformation (Figure 1C). While the precise mechanism by which DOT1L contributes to gene activation is not fully understood, DOT1L inhibits recruitment of a repressive SIRT1 and SUV39H1 complex, thus maintaining an open chromatin state permissive for gene expression (56). Various *in vitro* and *in vivo* experimental systems have shown that DOT1L and the interaction between DOT1L and MLL fusion partners is critical for development of leukemia in patients with MLL translocations (57–59).

The S-adenosyl methionine-competitive DOT1L inhibitor pinometostat (EPZ-5676) displays great specificity for DOT1L over other histone methyltransferases (60–63). Preclinical studies revealed that DOT1L inhibition specifically reduces H3K79 methylation and expression of MLL target genes leading to reduction of proliferation and viability as well as increased differentiation of leukemia cells both *in vitro* and *in vivo* (61). Phase I clinical studies of single agent pinometostat in adults (NCT01684150) and children (NCT02141828) with advanced or relapsed/refractory *MLL*-rearranged acute leukemia have recently been completed (64) (Table 1). Despite its limited pharmacokinetics, continuous intravenous administration was sufficient to decrease H3K79 methylation levels and expression of *HOXA9* and *MEIS1* in individual patients (64). However, only 2 out of 51 adult patients exhibited a clinical response (64) and no objective responses were reported in children (65). These somewhat disappointing results could perhaps be explained by the heterogeneity of MLL fusion proteins which may be differentially sensitive to DOT1L inhibition, uncertainties about optimal dosing, and biological discrepancies between enrolled patients and the preclinical models used to evaluate the effect of pinometostat. Further evaluation of pinometostat in combination with conventional chemotherapy in *MLL*-rearranged acute leukemia is currently underway (NCT03724084).

### PRMT5

Arginine methylation is increasingly appreciated as an important post-translational modification involved in regulation of transcription and chromatin organization, RNA processing and DNA damage repair (66–68). Arginine methylation is catalyzed by a family of nine protein arginine methyltransferases (PRMTs). However, recent research has mainly focused on the type II protein arginine methyltransferases PRMT1 and PRMT5. PRMT1 promotes H3R4 methylation, which is associated with an active chromatin state at critical promoters during hematopoietic cell differentiation; it is essential for recruitment of the acetyltransferase EP300 (69). PRMT1 can also methylate RUNX1, a key transcription factor required for definitive hematopoiesis, myeloid differentiation, and lymphocyte development (70). PRMT5 modifies H4R3, H2AR3, and H3R8, marks which are associated with transcriptional repression (71–75), and also targets multiple non-histone proteins including components of the spliceosome, PIWI proteins, EGFR, E2F1, TP53, and the NFκB subunit p65 (76–81). Multiple studies have implicated PRMT family members in cancer (82). Importantly, CRISPR-Cas9 screens in MLL-rearranged AML mouse models defined *PRMT1* and *PRMT5* as essential genes and consequently potential targets in this type of leukemia (83). Although PRMT1 is necessary for leukemic transformation, it is not sufficient for MLL-translocation dependent transformation. PRMT1 needs co-recruitment of KDM4C, an H3K9 demethylase, to regulated expression of MLL-fusion targets, such as HOXA9 (84, 85). Deletion or pharmacologic inhibition of both KDM4C and PRMT1 inhibits transcription and leukemic capacity of MLL fusions *in vitro* and *in vivo* (84). In keeping with this, conditional deletion or small molecule inhibition of PRMT5 impaired leukemia development and implicated PRMT5 as an enforcer of the leukemic differentiation block (86).

The first PRMT5 inhibitor to enter clinical trials is GSK3326595, a peptide competitive, S-adenosyl methionine-uncompetitive inhibitor. Although the mechanism of action has not been completely determined, GSK3326595 binds to the substrate recognition site of PRMT5 to inhibit methyltransferase activity and this is associated with decreased proliferation of leukemic cells (87). Results from phase 1 clinical trials in subjects with solid tumors and NHL (NCT02783300) as well as relapsed and refractory myelodysplasia, chronic myelomonocytic leukemia and secondary AML with a low proliferation fraction (NCT03614728) are awaited (Table 1).

### BRD4/BET Proteins

BRD4 is a member of the Bromodomain and Extra-Terminal motif (BET) family of proteins, and was identified as a potential cancer therapeutic based on results of a genome-wide shRNA screening in MLL-dependent AML cells (88, 89). BRD4 contains a bromodomain which enables its binding to acetylated lysines in histone H3 and H4 (Figure 1C). As a result, BRD4 is bound to active enhancers genome-wide, but is particularly associated with super-enhancers which are regions characterized by unusually high levels of H3K27 acetylation. BET proteins have been found to maintain aberrant chromatin states in AML and other hematologic malignancies (88, 90–92) in particular through regulation of *MYC* expression (88). Genetic and shRNA-mediated silencing of *BRD4* in MLL-AF9 driven leukemia models not only resulted in the removal of BRD4 from super-enhancers, including the MYC enhancer (93), but also in differentiation of leukemia cells and decrease of leukemogenic potential *in vitro* and *in vivo*.

The inhibition of BET proteins with preclinical inhibitors, such as JQ1 showed promising results in several studies in AML cell lines and *ex vivo* patient samples or mouse models, in particular in specific subtypes with *MLL* rearrangement, or those with mutations in *NPM1, FLT3* or *IDH2*, or *EVI1* overexpression (89, 94–97). Based on these observations, clinical trials of a number of BET inhibitors in AML, lymphoma and solid tumors were initiated including FT1101 (NCT02543879), MK8628 (NCT02698189), RO6870810 (NCT02308761), GSK525762 (NCT01943851, EudraCT 2013-000445-39), ABBV-744 (NCT03360006), ABBV-075 (NCT02391480), CPI-0610 (NCT02158858), and INCB054329 (NCT02431260) (Table 1). Few trial results have been published, but so far their clinical activity as single agents for relapsed or refractory AML appears in the main modest, despite the initial excitement arising from preclinical study data. Alternative combinatorial approaches may still capitalize on the clinical potential of these inhibitors, and studies are underway (98, 99). It is however noteworthy that some complete remissions were seen in a phase 1 study of MK8628 (OTX015), an analog of JQ1 (NCT01713582) (99).

## Emerging Therapeutic Options for AML

### CREBBP and EP300

Lysine acetyltransferases (KATs) and histone deacetylases (HDACs) catalyze the dynamic and reversible acetylation of histone and non-histone proteins, and are involved in major epigenetic regulatory mechanism of gene transcription (100) in normal hematopoiesis as well as various malignancies. While HDAC inhibitors have been investigated quite extensively in patients with myeloid malignancies, and without much success, development of KAT inhibitors has been largely neglected. The lysine acetyltransferase paralogs CREBBP (CBP; KAT3A) and EP300 (KAT3B) are transcriptional co-activators regulating a variety of cellular processes. Studies in heterozygous and conditional knockout mice have shown that CREBBP is an essential regulator of HSC differentiation, quiescence, apoptosis and self-renewal (101).

CREBBP and EP300 have been implicated in the development of various malignancies, including solid tumors and hematologic diseases (102). Indeed they are among the most frequently mutated KATs in blood cancers, in particular in lymphoma, with inactivating mutations mainly affecting the acetyltransferase domain. Importantly, CREBBP and EP300 are also found as oncogenic fusion partners of the histone acetyltransferase gene *MOZ* or *MLL* in leukemia (103, 104) (Figure 1D). In the MLL-CREBBP fusion, the bromo- and acetyltransferase domains of CREBBP are retained and are required for transformation. Additionally, downstream of fusion oncoproteins, recruitment of CREBBP and EP300 to chromatin binding sites for the transcription factor MYB is essential for the differentiation block in leukemias initiated by a range of fusions including AML1-ETO and MLL-AF9 (105, 106). In AML1-ETO AML, EP300 interacts directly with the AML1-ETO protein to regulate transcription of AML1-ETO target genes that are important for leukemic stem cells proliferation and self-renewal (107). CREBBP and EP300 have also been associated with transcriptional activation in collaboration with other leukemogenic proteins, such as NUP98-HOXA9 (108). Thus, there is quite some evidence that CREBBP and EP300 serve important roles in leukemic hematopoiesis and that their therapeutic targeting might be beneficial.

The multidomain organization of CREBBP and EP300 paralogs has prompted several inhibitor development projects. The most potent KAT inhibitors developed so far have been C646 (109) and I-CBP112 (110), an acetyl-lysine competitive protein–protein interaction inhibitor. Both induced differentiation and impaired leukemia-initiating potential in AML1-ETO^+^ or MLL-AF9^+^ AML cells *in vitro* and *in vivo*. More recently, a CREBBP and EP300 bromodomain inhibitor (CCS1477) has been demonstrated to have potent anti-proliferative and pro-differentiation activity in AML cell lines and primary patient samples (111); a first-in-human phase 1 study has commenced in castration-resistant prostate cancer (NCT03568656) and a related study will shortly commence in patients with multiple hematologic malignancies, including AML.

### MEN1 and WDR5

Recent reports have demonstrated that MLL-driven gene expression is dependent on the interaction of MLL with menin (MEN1) (112). MEN1 serves as an adaptor for the interaction of MLL with LEDGF, a protein that tethers the MLL complex to chromatin (112). This interaction is also crucial for leukemic transformation, proliferation, and expression of leukemia associated genes including the HOXA-cluster and *MEIS1* (113) (Figure 1E). Interestingly, while the interaction of MEN1 with MLL1 is not essential for normal hematopoiesis (114), genetic disruption of the MEN1-MLL fusion protein interaction abrogates the oncogenic properties of MLL fusion proteins and blocks the development of AML *in vivo* (113), highlighting this interaction as an attractive therapeutic target to develop targeted drugs for MLL leukemia patients. Another potentially interesting approach to disrupt the MLL-fusion complex is inhibition of the interaction between WD repeat domain 5 (WDR5) and MLL1. WDR5 directly interacts with SETD1A, SETD1B or one of four homologous MLL methyltransferases (115, 116), which are components of the MLL methyltransferase complex. This interaction is required for the catalytic activity of the enzymes and is responsible for H3K4-specific methylation, a histone mark generally associated with transcriptional activation.

Several small molecules and tool compounds targeting the interactions of components of the MLL complex have been recently developed. Inhibitors targeting MEN1-MLL have been shown to reverse *HOXA* and *MEIS* gene expression, thereby releasing the differentiation block associated with MLL-rearranged leukemia (117–121). Similarly MM401 and OICR9429, two compounds which disrupt the MLL-WDR5 interaction, inhibit the proliferation of AMLs harboring *MLL* translocations (122) or CEBPA mutations (123). However, limited bioavailability and efficacy *in vivo* is still an important hurdle to overcome.

## Conclusion

Epigenetic regulators are central players in the initiation and maintenance of hematopoietic malignancies, an observation which has resulted in myriad opportunities for development of targeted therapies. In particular, where epigenetic mechanisms are specifically disordered in malignant but not normal blood cells, there exists the potential for a significant therapeutic window. Along these lines, there has been significant progress in understanding the role of epigenetic modifications and their modifiers in cancer in general and in AML in particular. The discovery and development of small-molecule inhibitors targeting certain epigenetic regulators has already led to opportunities for clinical trial evaluation and potential patient benefit; impending trial results will inform on efficacy and safety. While the evaluation of many of these compounds for their single agent activity is an essential first step, it will be critical to test efficacy in combination with either standard-of-care chemotherapies or novel therapeutics, to determine their optimal role in the treatment of leukemia. In time, personalization of therapeutic regimens according to patient cytogenetics and molecular mutations may become of essential importance. A final point is that the interdependence of cancer epigenetics and immunological responses has to be taken in consideration. In the setting of leukemia, this includes the therapeutic modality of allogeneic stem cell transplantation. It is clear that epigenetic therapies can induce cellular responses in tumor cells that interact with the immune system and which may contribute to their efficacy; for example, effects of adoptive immunotherapies and immune checkpoint inhibitors can be potentiated by epigenetic therapies (124). As such, the combination of immunotherapies and epigenetic therapies also holds potential promise for the development of additional therapeutic options in AML.

## Author Contributions

BW and TS conceived and wrote the manuscript.

### Conflict of Interest Statement

The authors declare that the research was conducted in the absence of any commercial or financial relationships that could be construed as a potential conflict of interest.
